# Suppression of tumor growth and apoptosis induction by pomegranate seed nano-emulsion in mice bearing solid Ehrlich carcinoma cells

**DOI:** 10.1038/s41598-023-32488-6

**Published:** 2023-04-04

**Authors:** Hanan R. H. Mohamed, Fadi S. A. Tulbah, Akmal A. El-ghor, Shaymaa M. Eissa

**Affiliations:** Zoology Department, Faculty of Science, Cairo University, Giza, Egypt

**Keywords:** Molecular biology, Natural hazards, Health care

## Abstract

Despite the high antioxidant and penetration ability of pomegranate seed oil (PSO), the in vivo antitumor activity of PSO nano-emulsion has not been well investigated. Therefore, this study was undertaken to estimate the antitumor activity and safety of PSO nano-emulsion in mice bearing Ehrlich solid carcinoma cells. For tumor inoculation, about 2 × 10^6^ viable Ehrlich tumor cells (200 µl) were implanted intramuscularly in the left thigh of hind leg. Once a solid tumor appears on the 10th day of transplantation; the mice were randomly divided into five groups (5 animals/group). Characterization of the PSO nano-emulsion using a Zeta sizer Malvern instrument and transmission electron microscope (TEM) revealed that the PSO nano-droplets were well dispersed with an average particle size of 8.95 nm and a spherical shape. Treatment with PSO nano-emulsions caused a significant reduction in the tumor size and weight, in a dose dependent manner, compared to tumor control group. Marked dose dependent elevations in the DNA damage level together with significant increases in the tumor suppressor p53, Bax and Caspase genes and reductions in the anti-apoptotic Bcl2 gene were also observed in the tumor tissue of mice given PSO nano-emulsions. Histological examination also revealed apoptosis and necrosis of tumor cells and tumor infiltration with inflammatory cells after PSO nano-emulsion treatment. However, high DNA damage was noticed in the liver and kidney tissues of mice given the highest dose of PSO nano-emulsion (400 mg/kg). Therefore, we concluded that PSO nano-emulsion exhibited a potent antitumor activity through induction of DNA breaks that triggers apoptosis of tumor cells but the highest dose caused genotoxicity to liver and kidney tissues, thus it is recommended to use doses lower than 400 mg/kg of PSO nano-emulsion as an alternative drugs for chemotherapy.

## Introduction

Cancer is one of the main causes of death worldwide according to the World Health Organization. There are several problems associated with the use of cancer chemotherapy including: severe systemic toxicity due to lack of selectivity to cancer cells and limited available dose targeting cancer cells. Because of the problems associated with conventional cancer treatment, increased attention has been paid to developing new treatment^1–4^.

Pomegranate fruit is known as tree *Punica granatum* and grown in the Mediterranean region. Over the past years, many scientific researches have been investigated the traditional remedy applications of the different anatomical compartments of pomegranate fruit^5^. It has been found that pomegranate seed contains vitamin E, sterols and punicic acid, in good quantities^6^. Pomegranate seed oil (PSO) has been shown to be effective in reducing neurodegenerative diseases, colon cancer, diarrhea, ulcers, cardiovascular protection, oral hygiene, hyperlipidemia and breast cancer^7–10^. Several other studies have highlighted the potential pleiotropic effects of PSO such as antioxidant, anti-inflammatory, antiangiogenic, immunomodulatory function, suppressing chemically induced carcinogenesis and anti-cancer activity^8,11–14^.

Many studies have also discovered the anti-tumor effects of PSO and its ability to treat cancer diseases, for example Jeune et al.^15^ reported the ability of PSO to treat breast cancer in human breast cancer cell (MCF-7) line. In a similar study by Nallanthighal et al.^16^, it was found that pomegranate extract had the ability to suppress cancer stem cells due to inhibition of epithelial-to-mesenchymal transition. Promising antitumor activity of PSO has been shown by the reported ability of PSO to inhibit various tumor cells such as breast, prostate and skin tumors, disrupt cell cycle by inducing apoptosis and thus, reducing the tumor growth^17–20^.

In in vivo a study has shown that PSO had the ability to decrease malondialdehyde level, DNA fragmentation, caspase-3 and Glutathione activities in diethylnitrosamine and phenobarbital-induced hepatic injury in male rats^21^. In vitro exposure of PSO on human skin cells as a cosmeceutical source had been evaluated. The study found that PSO promote proliferation, procollagen synthesis and inhibit matrix metalloproteinase-1 production on skin repair^18^.

Moreover, the kinetic stability of nano-emulsions and their small droplet size (100–500 nm) prevent instability phenomena generated by creaming, coalescence and/or sedimentation^22,23^. The smaller droplet size of nano-emulsions makes the formulations less viscous and more transparent than traditional emulsions^24^. Furthermore, due to droplet size reduction, nano-emulsions have been studied to increase drug bioavailability, enhance skin penetration, improve water solubility of lipophilic compounds and to ameliorate stability of denatured materials.

Ehrlich's ascites carcinoma (EAC), an undifferentiated carcinoma, is characterized by high transplantability, rapid proliferation, short lifespan, 100% metastasis and no tumor-specific transplant antigen. Ehrlich solid carcinoma develops from EAC subcutaneous inoculation because it resembles human tumors and is therefore used in many studies as an experimental model to study the antitumor effect of drugs or natural compounds^25–27^.

Based on the aforementioned very attractive therapeutic and nutritional properties of nan-emulsions together with the demonstrated toxic side effects of the used anticancer drugs, this study was undertaken to explore the possible antitumor activity of PSO nano-emulsion in mice. Tumor weight and volume were measured during the experimental period. Comet assay was conducted to assess DNA breaks, while the expression levels of apoptotic and anti-apoptotic genes were measured using Real-Time PCR. Immuno-histochemical of p53 and Caspase proteins along with histopathological examination were also performed. Moreover, the level and activity of biochemical markers of oxidative stress were measured.

## Materials and methods

### Animals

Adult (6-week-old) C57BL/6 female mice weighing 25–30 g were purchased from the National Research Center (NRC, Giza Egypt) Animal House, and acclimated for 1 week prior to experiments in a temperature-controlled environment (22–25 °C), humidity, and a 12-h light/dark cycle. Mice were housed in polypropylene cages (5 animals per cage) and allowed a free standard laboratory diet.

### Ethical consideration

The current study was performed according to ARRIVE guidelines and all experimental procedures were conducted in accordance with international guidelines for the care and use of laboratory animals. The experimental animal protocol was approved by the Institutional Animal Care and Use Committee (CU-IACUC) of the Faculty of Science, Cairo University with approval number CU/I/F/5/20.

### Chemicals

The Pomegranate seed oil (PSO), dimethylsulfoxide (DMSO), 1-1-diphenyl-2-picrylhydrazyl (DPPH), absolute ethanol, formaldehyde, tris-(hydroxymethyl)-amino methane (Tris-base), ethylenediaminetetracetic acid disodium salt (Na2EDTA), triton X-100, and ethidium bromide (EtBr) were purchased from Sigma-Aldrich (USA). Kits for all biochemical parameters were purchased from Bio-diagnostic Company (Giza, Egypt).

### Preparation of PSO nano-emulsion

PSO nano-emulsions were prepared by auto-emulsification according to the method described by Ferreira et al.^28^ Briefly, PSO (3.0% (w/v)-NE PSOB) and Span 80 (0.077 g) were dissolved in acetone (50 ml). After 60 min with moderate stirring, the organic phase was added into Tween 80 (0.077 g) aqueous phase (50 mL). The O/W emulsion was formed instantly by diffusion of the organic solvent into the aqueous phase, forming the nano-droplets. The magnetic stirrer was kept for 10 min and then the organic solvent was discarded by evaporation under reduced pressure to achieve a final volume of 10 ml.

### Characterization PSO nano-emulsion

Average droplet size and polydispersity index (PDI) (n = 3) were measured at 25 °C by photon correlation spectroscopy (Zeta sizer Nanoseries, Malvern instruments, UK) after diluting the samples in ultrapure water (1:500). Zeta potential (ZP) was measured using the same instrument after diluting samples in 10 mM NaCl (1:500). The pH values of the nano-emulsions were determined by direct immersion of the electrode of a calibration potentiometer into the formulations. The measurements were carried out at room temperature (25 ± 2 °C). The morphology of nano-emulsions droplets was also studied by imaging these droplets using transmission electron microscope (TEM).

### Acute toxicity test

Acute toxicity assay was performed to detect the appropriate test dose of PSO nano-emulsion using OECD-420 guidelines. Ten female mice were randomly divided into control and test groups each containing five animals. Mice in the control group were given deionized distilled water, while, mice of the test group were intraperitoneally (i.p) injected with PSO nano-emulsion at dose levels of 2000 mg/kg. All mice were then carefully observed for 24 h after nano-emulsion injection until the end of the 14-day experimental period for signs of toxicity, morphological behavior and mortality^29^. The studied doses of PSO nano-emulsion were detected as 20%, 10% and 5% of safety test dose obtained from the OECD test.

### Experimental design and tumor inoculation

Thirty females were randomly divided into the healthy negative control group (group 1) and five tumor-bearing groups (groups 2–6), five mice per group. For tumor inoculation, the Ehrlich ascites tumor, obtained from Murine Ehrlich ascites carcinoma (ESC) bearing mouse and derived from a spontaneous murine mammary adenocarcinoma, is maintained in the ascetic form by passages in Swiss mice by weekly intraperitoneal transplantation of 2–3 × 10^6^ tumor cells. With a sterile 3 ml syringe the ascetic fluid was collected from the peritoneum carefully and tumor cell were counted using a Neubauer hemocytometer to ensure cells viability of greater than 95%. Mice were then implanted with 200 µl of Ehrlich tumor cell suspension (containing about 2 × 10^6^ viable cells) intramuscular in the thigh of the left hind leg. Once solid tumor appeared on day 10 of implantation; 25 mice were randomly divided into five groups (5 animal/ group).

Group 1: Healthy negative control group without tumour in which mice given deionized dist. water three times per week for 2 weeks.

Group 2: Ehrlich solid carcinoma bearing mice given deionized dist. water three times per week for 2 weeks.

Group 3: Ehrlich solid carcinoma bearing mice injected intraperitoneally with Doxorubicin (2 mg/kg) twice per week^30^ for 2 weeks.

Group 4: Ehrlich solid carcinoma bearing mice treated intraperitoneally with 100 mg/kg (5% of the determined safe dose) of PSO nano-emulsion three times per week for 2 weeks.

Group 5: Ehrlich solid carcinoma bearing mice treated intraperitoneally with 200 mg/kg (10% of the determined safe dose) of PSO nano-emulsion three times per week for 2 weeks.

Group 6: Ehrlich solid carcinoma bearing mice treated intraperitoneally with 400 mg/kg (5% of the determined safe dose) of PSO nano-emulsion three times per week for 2 weeks.

### Tumor measurements

Tumor volume was measured every four days using a Vernier caliper based on equation: tumor volume (mm^3^) = length (mm) × (width (mm))^2^/2. Individual relative tumor volume (RTV) was measured using the formula Vx/V1, where Vx is the volume in mm3 at a given time and V1 at the start of the treatment. Tumor growth inhibition (%TGI) was also calculated using the equation 100 − (T/C × 100), where T is the mean RTV of the treated tumor and C is the mean RTV in the control group at the same point time^31^.

### Sample collection

After 24 h of the last administration of the tested substances, all mice were euthanized using sodium Isoflurane (1–4%) and sacrificed by cervical dislocation. The liver, kidney and solid tumor in the left thigh of mice were immediately dissected out and stored at – 80 °C for biochemical and molecular studies. Small portions of these tissues were immediately preserved in 10% formalin for histopathological and immuno-histochemical studies.

### Estimation of DNA damage using comet assay

The DNA damage extent in the kidney, liver and tumor tissues was measured for all six groups using the alkaline comet assay^32^. A mixture of cell cells suspensions (10 µl) and 05% low melting agarose (75 µl) was sprayed onto a clean slide coated with a thin layer of normal melting agarose (1%). Slides with solidified gel were immersed in cold lysis buffer and left in the dark for 24 h. After incubation of slides in freshly prepared alkaline electrophoresis buffer for DNA unwinding, denaturated DNA was electrophoresed for 35 min at 25 V and 300 mA. The DNA was then neutralized, and fixed in absolute cold ethanol. Prior imaging, slides were stained with ethidium bromide and fifty comet cells per animal were scored using a TriTek CometScore ™ Freeware v1.5 scoring software. Tail length in px, %DNA in tail and tail moment were used as indicators for DNA damage.

### Expression levels of P53, Bax and Bcl2 genes

The entire cellular RNA was extracted from tumor tissues of mice bearing ESC based on the instructions described in Thermo Scientific Gene JET™ RNA purification kit (Thermo Scientific, USA). According to the instructions described in RevertAid First Strand cDNA Synthesis Kit (Thermo Scientific, Waltham, MA, USA) 5 µg of the purified RNA was then reversely transcribed into complementary DNA (cDNA). The primers required for amplification of p53, Bax, Bcl2 and also GAPDH genes (Table 1) were designed using NCBI Primer blast and synthesized by Invitrogen Company (Carlsbad, CA, USA).

**Table 1:** Primers sequences used in quantitative real time PCR assay.

Genes	Primers	Sequences	Bp
GAPDH	Foreword	Gtatcggacgcctggttac	128 bp
	Reverse	Cttgccgtgggtagagtcat	
Bax	Foreword	Gtctccggcgaattggagat	100 bp
	Reverse	Acccggaagaagacctctcg	
Bcl2	Foreword	Catcgccctgtggatgactg	95 bp
	Reverse	ggccatatagttccacaaaggc	
P53	Foreword	Cccctgtcatcttttgtccct	137 bp
	Reverse	Agctggcagaatagcttattgag	

Amplification of the p53, Bax, and Bcl2 genes was performed using 7500 Fast systems (Applied Biosystem, Foster City, CA, USA): For each PCR reaction, a mixture of total volume 25 µl contained 12.5 µl of SYBER Green master (Thermo Scientific Maxima SYBR Green qPCR Master Mix), 2.5 µl synthesized cDNA, 1 μl of forward and reverse primers^33^ and 8 µl PCR grade water. The prepared 25 µl PCR mixture contained 12.5 µl master mix (Thermo Scientific Maxima SYBR Green qPCR Master Mix), 2.5 µl sample (cDNA), 1 µl forward and 1 µl reverse primers and 8 µl PCR-grade water. PCR samples were initially heated at 95 °C for 15 min and 40 cycles of denaturation at 95 °C for 15 s, annealing and elongation at 60 °C for 1 min was conducted. For normalization, the Glyceraldehyde 3-phosphate dehydrogenase (GAPDH) gene was amplified as an endogenous housekeeping gene. The expression levels of the studied genes p53, Bax and Bcl2 genes were quantified using the comparative Ct (∆∆CT) method.

### Histopathological examination

For histopathological studies, fresh portions of the muscle tissues were immediately immersed in 10% buffered formalin. Fixed tissues were embedded in paraffin wax and sectioned using microtome into thin sections with 5 µm thickness, then stained with hematoxylin and eosin. The stained sections were photographed and examined under the light microscope.

### Immuno-histochemical study

Immuno-histochemical analysis of p53 and caspase-3 was performed^34^ using the streptavidin–biotin method by Histostain-plus assay. 5 mm thick paraffin tumor sections were dewaxed in xylene, and rehydrated through an ascending series of alcohols. Non-specific binding was blocked using non-immune serum (10%). Tissues were incubated with p53 antibody (Code RMPD 016, ready-to-use rabbit monoclonal antibody, DBS, CA, USA) or Caspase-3 antibody (Code MS1770’R7, ready-to-use antibody, Thermoscientific, CA, USA) diluted 1:50 with TBS for 2 h. For negative control, tissues were incubated with TBS only. Tissue endogenous peroxidase activity was blocked using 3% hydrogen peroxide (Bio Genex, San Ramon, CA, USA). To visualize tissues were incubated with 100 ml of horseradish peroxidase labeled mouse secondary antibody, and then the DAB chromogen was added. Sections were counterstained with hematoxylin, dried, mounted and examined using a light microscope (Zeiss) to assess p53 immunostaining. Nuclei positive for p53 accumulation were stained brown.

### Measurement of oxidative stress biomarkers

The level of malondialdehyde (MDA), lipid peroxidation end product, was measured in the tumor tissue homogenate based on the reaction of MDA with thiobarbituric acid at low PH^35^. On the other hand, the level of antioxidant reduced glutathione (GSH) and activities of the antioxidative enzymes catalase (CAT) and superoxide dismutase (SOD) were determined based on the previously described protocols^36–38^.

### Statistical analysis

The data were analyzed^39^ using the Statistical Package for Social Sciences (SPSS) version 22. According to the Kolmogorov-Smirnova and Shapiro–Wilk tests, the data were normally distributed within the groups. Accordingly, standard analysis was applied to the statistical analysis of the data. A one-way analysis of variance (ANOVA) was used to study the effect of treatment on the variables studied. Two-ways ANOVA was performed to study the effect of treatment and time and their interaction on the studied parameters. Duncan's test was used to study the similarity between the studied groups. Regression analysis and correlation coefficient were used to fit the relationship between the nano concentration or time and the studied parameters. Data are presented as mean ± standard error of the mean.

## Results

### Characterization of PSO nano-emulsion

**Figure 1:**
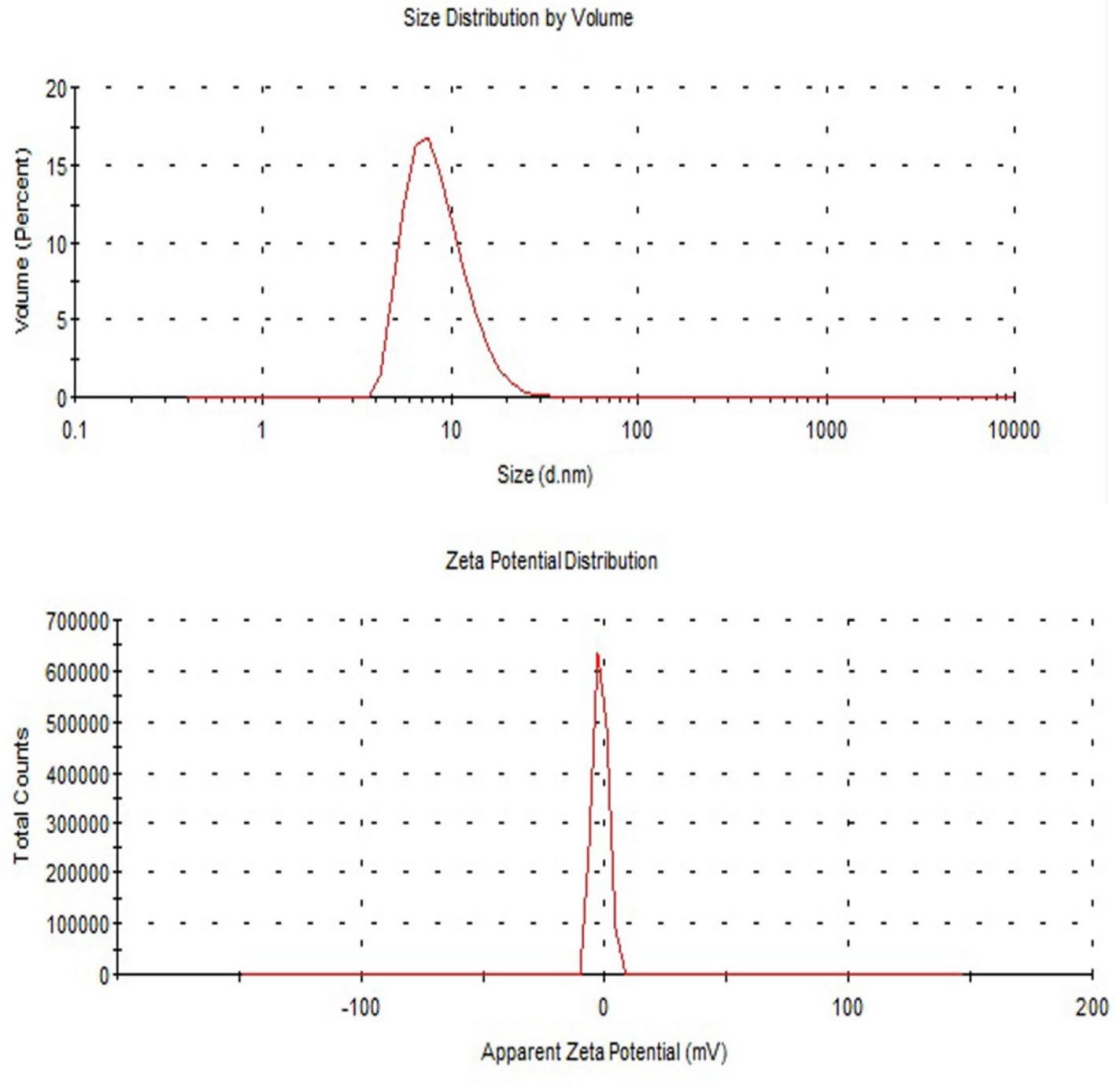
Size distribution and zeta potential distribution of PSO nano-emulsion.

**Figure 2:**
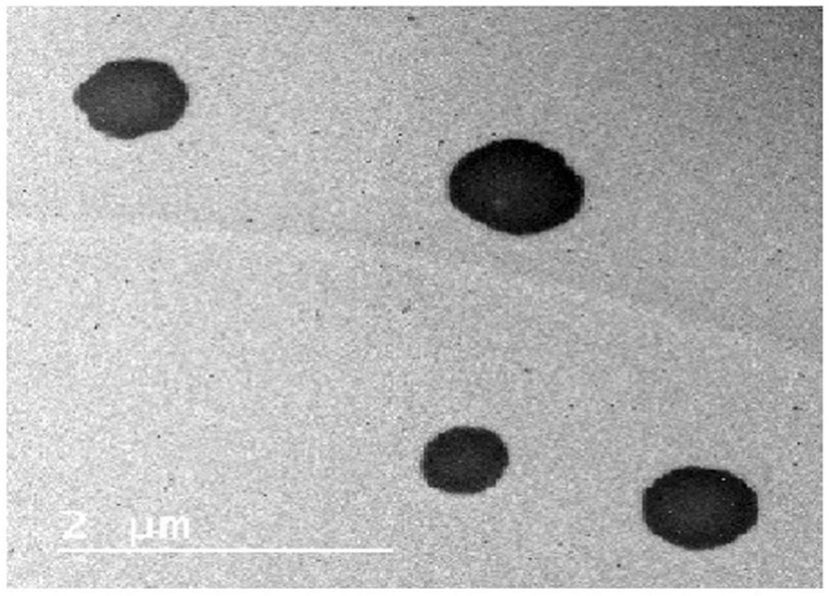
Transmission electron microscopy (TEM) image of PSO nano-emulsion.

Screening the hydrodynamic droplets' distribution of the PSO nano-emulsion revealed that PSO nano-droplets are well distributed and separated with an average droplets' size of 8.95 nm and polydispersity index (PdI) of 0.240 (Fig. 1). Imaging of PSO nano-droplets using TEM also showed that PSO nano-droplets are well scattered and have a spherical shape as seen in Fig. 2.

### Acute toxicity test

Observation of mice given PSO nano-emulsion at a dose level of 2000 mg/kg revealed that all mice were still healthy and showed no signs of toxicity during the first 48 h of PSO nano-emulsion treatment until the end of the fourteen days. Therefore, the half lethality dose (LD50) of PSO nano-emulsion was considered to be above 2000 mg/kg according to the OECD-420 guidelines, and the studied doses of PSO-nano-emulsion were calculated in this study as 5%, 10% and 20% of the LD50 Obtained from acute toxicity test, they are 100, 200 and 400 mg/kg body weight.

### Tumor size and weight

**Figure 3:**
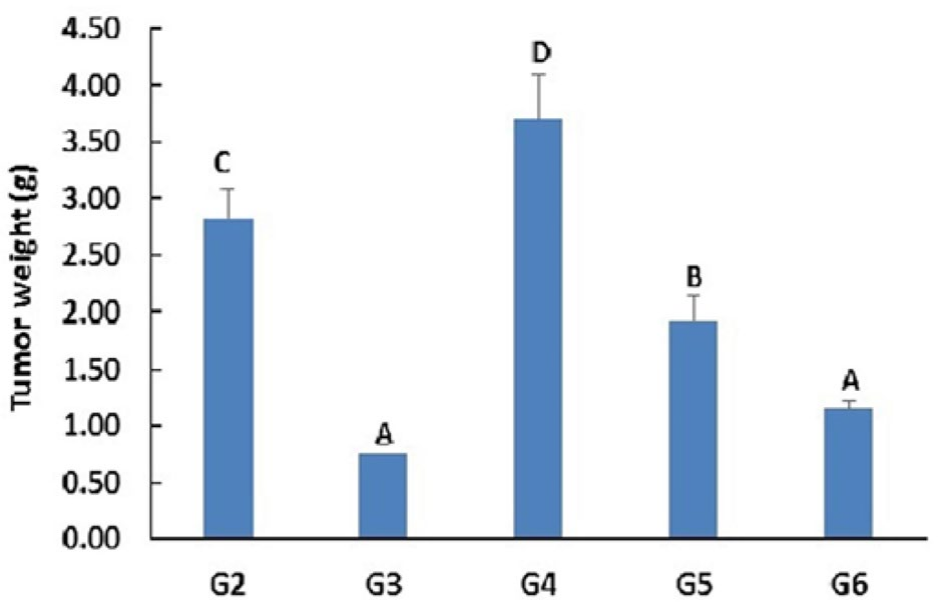
The weight of tumor (g) of tumor bearing groups. Data is displayed as mean ± standard error. G2: tumor control group, G3: doxorubicin-treated group, G4–G6: groups treated with 5, 10 & 20% of PSO nano-emulsion LD50, respectively. Means marked with the same superscript letters are insignificantly (*p* > 0.05) different, whereas those marked with different ones are significantly (*p* < 0.05) different.

**Figure 4:**
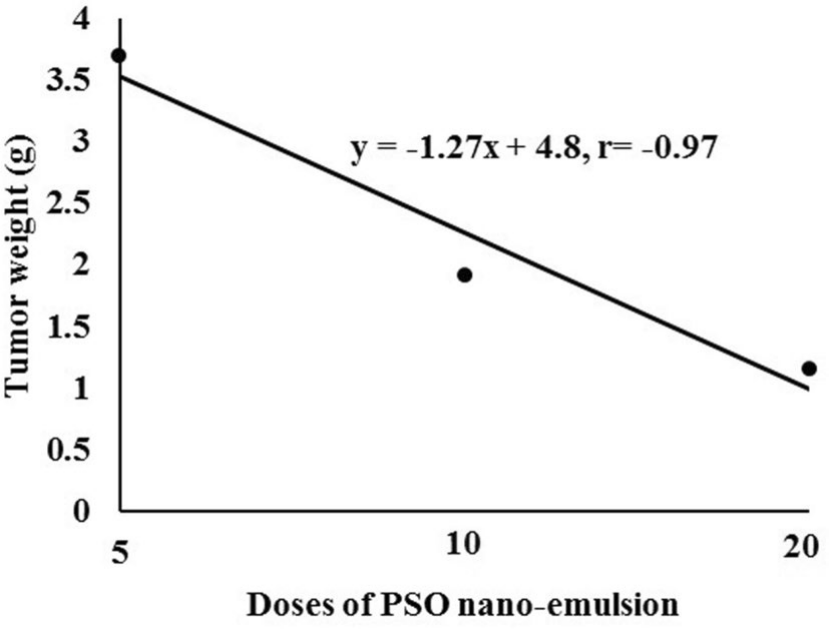
Regression analysis of tumor weight with the levels of PSO nano-emulsion different doses 5, 10 and 20% of PSO nan-emulsion LD50. r: Pearson’s correlation coefficient.

Monitoring of mice bearing tumor showed that tumor weight was significantly affected by PSO nano-emulsion treatment (Fig. 3). Compared with the tumor control group (G2 Group), the tumor weight of all the other experimental groups was markedly declined except for the remarkable elevation in mice when given 100 mg/kg b.w of PSO nano-emulsion (G4 Group) as display in Fig. 3. Regression analysis and correlation coefficient discovered that tumor weight was markedly decreased by increasing the doses of the given PSO nano-emulsion, in a dose-dependent manner (Fig. 4).

**Figure 5:**
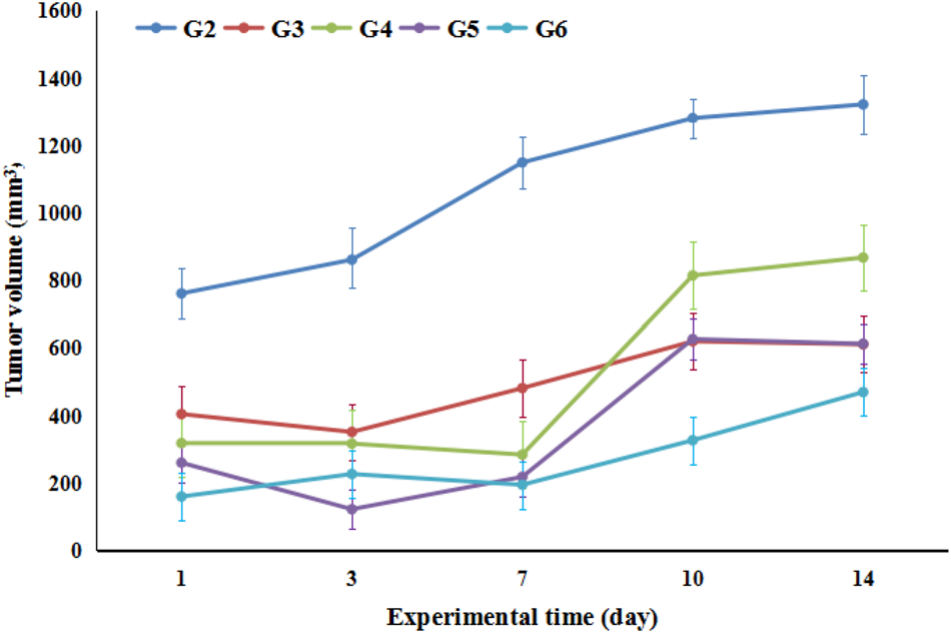
The tumor volume of tumor bearing experimental groups. Data is displayed as mean ± standard error. G2: tumor control group, G3: doxorubicin-treated group, G4–G6: groups treated with 5, 10 & 20% PSO nano-emulsion, respectively.

On the other hand, the tumor volume at the 3rd and 7th days in most of the experimental groups showed insignificant change, followed by marked elevations at the rest of the experimental periods as compared to the 1st day (Fig. 5). In comparison to tumor control group (G2 Group), the tumor volume of mice given 100 mg/kg of PSO nano-emulsion (Group G3) was significantly reduced, at all the experimental intervals. At most intervals, the tumor volume of mice-treated with nano was similar to those of G3 group treated with doxorubicin except for remarkable reductions in mice treated with PSO nano-emulsion at a dose level of 400 mg/kg (Group G6), at the 3rd, 7th and 10th days (Fig. 5). Two-ways ANOVA revealed that tumor volume was significantly affected by the type of treatment, experimental time and their interaction (Fig. 5). Although, strong negative correlations were recorded between the treatment type and tumor volume, tumor volume was positively correlated with the experimental time (Table 2).

**Table 2:** Correlation coefficients between the tumor volume and treatment (r_Tr_) and experimental time (r_Ti_).

Time (day)	r_Ti_	Treatment	r_Tr_
1	0.963487	G2	− 0.98875
3	0.911812	G3	− 0.46489
7	0.866446	G4	− 0.96275
10	0.834918	G5	− 0.99127
14	0.915592	G6	− 0.98718

G2, Tumor control group; G3, doxorubicin-treated group; G4–G6, groups treated with 100, 200 or 400 mg/kg of PSO nano-emulsion (G4, G5, G6) respectively.

### Induction of DNA damage

Induction of DNA damage was assessed in all experimental groups using Comet assay. The Comet parameters tail length, %DNA in tail, tail moment and olive tail moment were presented in Table 4. In the kidney of the tumor control group (G2 group), the tail length and olive tail moment were remarkably higher, while, %DNA in tail, tail moment and olive tail moment in the liver of G2 group, were markedly lower than those in the kidney and liver tissues of the healthy negative control group (G1 group) as displayed in Table 3. On the contrary, the values of %DNA in tail and tail moment in the kidney tissue and the tail length in the liver tissue of the G2 group did not change significantly compared to the corresponding values in the G1 group (Table 3).

**Table 3:** Tail length (TL), %DNA, tail moment (TM) and olive tail moment (OTM) in the tumor, liver and kidney tissues of normal negative control group (G1), tumor control groups (G2), doxorubicin-treated group (G3) and groups treated with 100, 200 or 400 mg/kg of PSO nano-emulsion (G4, G5, G6) respectively.

Parameters	Tissue	Groups					
		G1	G2	G3	G4	G5	G6
TL	Tumour	–	2.70 ± 0.30^A^	7.14 ± 0.74^B^	6.27 ± 0.60^B^	6.60 ± 0.41^B^	6.86 ± 0.50^B^
	Liver	2.53 ± 0.36^A^	2.15 ± 0.32^A^	3.29 ± 0.45^AB^	4.25 ± 0.57^B^	3.30 ± 0.44^AB^	3.48 ± 0.41^AB^
	Kidney	2.42 ± 0.34^A^	4.25 ± 0.45^B^	2.51 ± 0.31^A^	2.27 ± 0.33^A^	2.35 ± 0.33^A^	3.17 ± 0.38^A^
%DNA	Tumour	–	0.89 ± 0.12^A^	3.74 ± 0.46^BC^	2.50 ± 0.34^AB^	12.08 ± 1.20^D^	5.40 ± 0.63^C^
	Liver	7.32 ± 0.89^B^	3.62 ± 0.33^A^	5.63 ± 0.53^AB^	3.92 ± 0.40^A^	3.88 ± 0.42^A^	14.48 ± 1.48^C^
	Kidney	2.91 ± 0.45^A^	4.19 ± 0.47^AB^	5.80 ± 0.64^B^	5.49 ± 0.68^B^	3.57 ± 0.40^A^	7.61 ± 0.90^C^
TM	Tumour	–	0.05 ± 0.02^A^	0.36 ± 0.08^BC^	0.22 ± 0.05^AB^	0.91 ± 0.12^D^	0.46 ± 0.08^C^
	Liver	0.41 ± 0.10^B^	0.06 ± 0.02^A^	0.26 ± 0.07^AB^	0.25 ± 0.06^AB^	0.17 ± 0.06^AB^	0.98 ± 0.18^C^
	Kidney	0.08 ± 0.02^A^	0.21 ± 0.05^A^	0.18 ± 0.04^A^	0.19 ± 0.05^A^	0.08 ± 0.02^A^	0.36 ± 0.09^B^
OTM	Tumour	–	0.10 ± 0.02^A^	0.38 ± 0.06^B^	0.33 ± 0.06^B^	0.78 ± 0.09^D^	0.40 ± 0.05^B^
	Liver	0.51 ± 0.07^B^	0.27 ± 0.03^A^	0.54 ± 0.08^B^	0.40 ± 0.05^AB^	0.28 ± 0.04^A^	0.87 ± 0.11^C^
	Kidney	0.16 ± 0.02^A^	0.35 ± 0.05^BC^	0.34 ± 0.04^BC^	0.39 ± 0.05^CD^	0.23 ± 0.04^AB^	0.51 ± 0.06^D^

Data is displayed as mean ± standard error.

In the same row, means marked with the same superscript letters are insignificantly (*P* > 0.05) different, whereas those marked with different ones are significantly (*P* < 0.05) different.

On the other hand, treatment with PSO nano-emulsion at the three dose levels of 100, 200 and 400 mg/kg (Groups G4, G5 and G6, respectively) significantly elevated tail length, % DNA in tail, tail moment and olive tail moment compared to their values in the tumor tissue of G2 group and also in mice injected with Doxorubicin (group G3) as displayed in Table 3. In the liver and kidney tissues of mice given 400 mg/kg of PSO nano-emulsion (G6 group), the %DNA in tail, tail moment and olive tail moment were meaningfully increased but tail length did not significantly changed compared to their values in G1 and G2 groups. Although, treatment with 100 or 200 mg/kg of PSO nano-emulsion (G4 and G5 groups, respectively) generally did not cause significant changes in the tail length, %DNA in tail, tail moment and olive tail moment compared to G1 and G2 groups, the hepatic tail length, renal %DNA in tail and renal olive tail moment were remarkably higher than their levels in the G1 group. The hepatic %DNA in tail in the G4 and G5 groups and hepatic olive tail moment of only G5 group was significantly lower than those in the G1 group as shown in Table 3.

**Figure 6:**
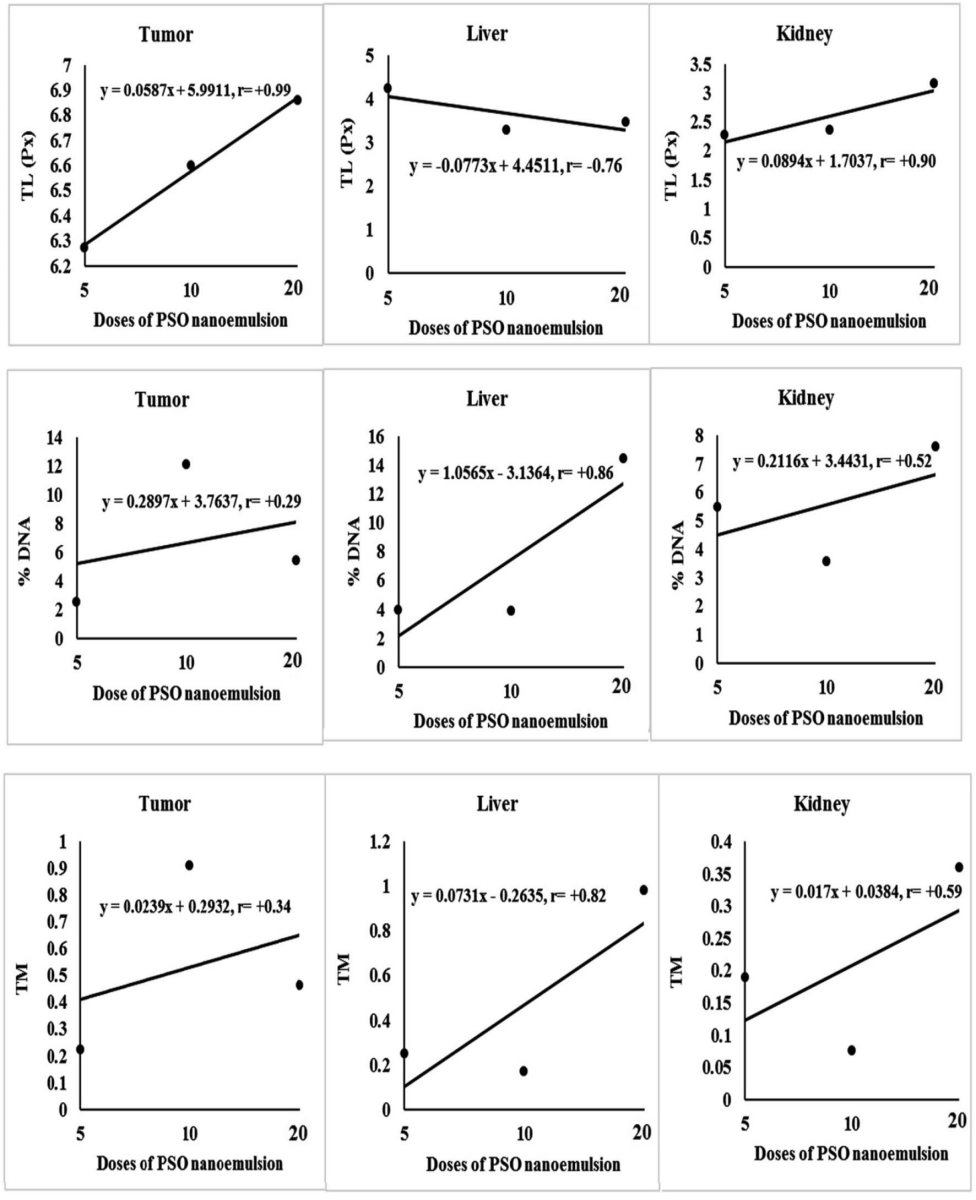
Regression analysis of tail length (TL), %DNA in tail and tail moment (TM) with the different doses of PSO nanoemulsion. r: Pearson’s correlation coefficient.

**Figure 7:**
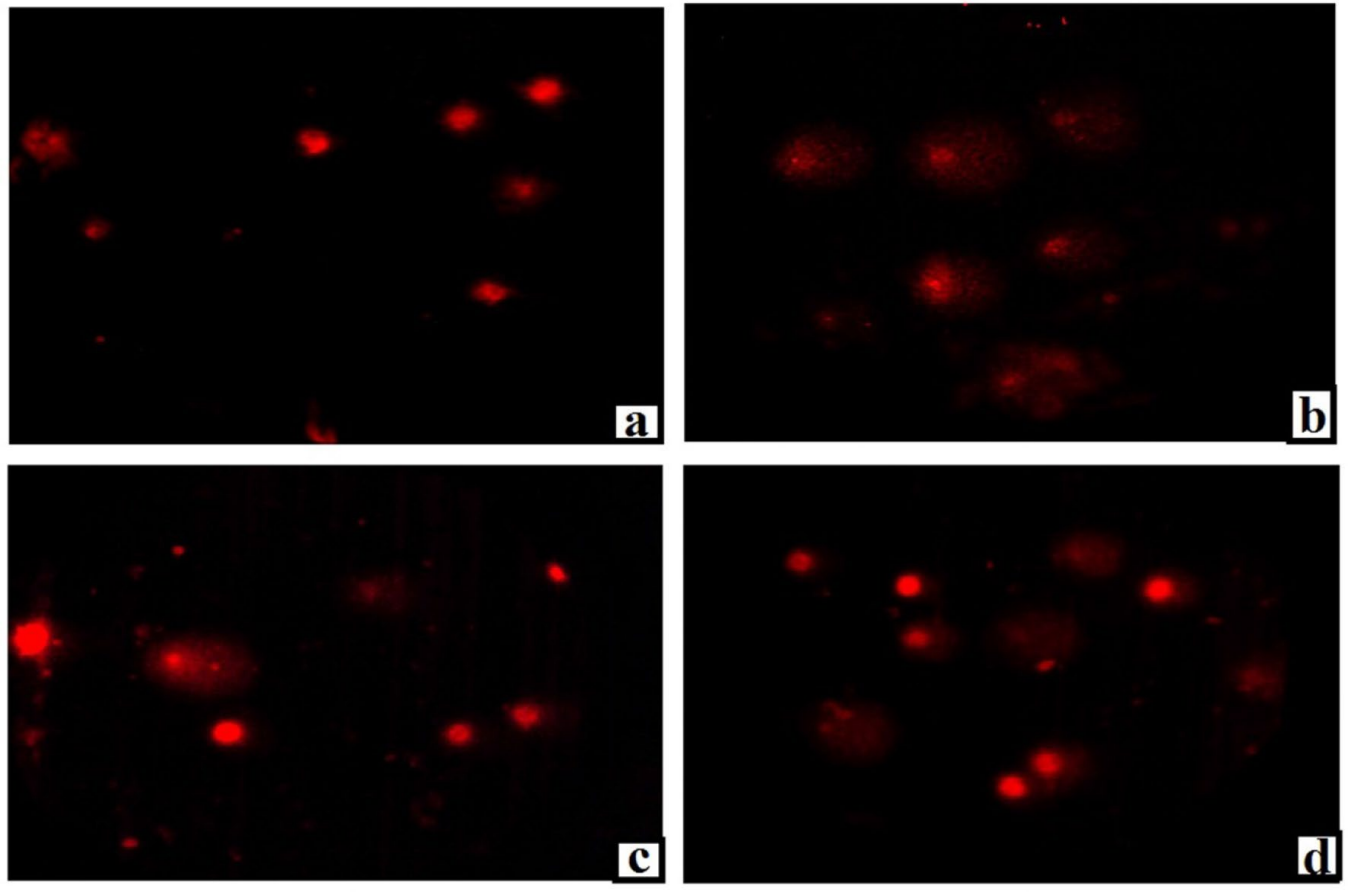
Representative examples for the scored Comet nuclei with intact DNA (**a**) and destructed DNA (**b**–**d**) with different grades of damage regardless of treatment. Magnification × 200.

Tail length and doses of PSO nano-emulsion were correlated positively in the tumor and kidney tissues whereas negatively in the liver tissue, while, positive relationships were obtained between the %DNA in tail and doses of PSO nano-emulsion in the liver and kidney tissues (Fig. 6). Direct relationships were also reported in the liver tissues between the doses of PSO nano-emulsion and tail moment (Fig. 6). Some of the scored comet nuclei with intact DNA and destroyed DNA with different degrees of damage are shown in Fig. 7.

### Expression levels of P53, Bax and Bcl2 genes

**Figure 8:**
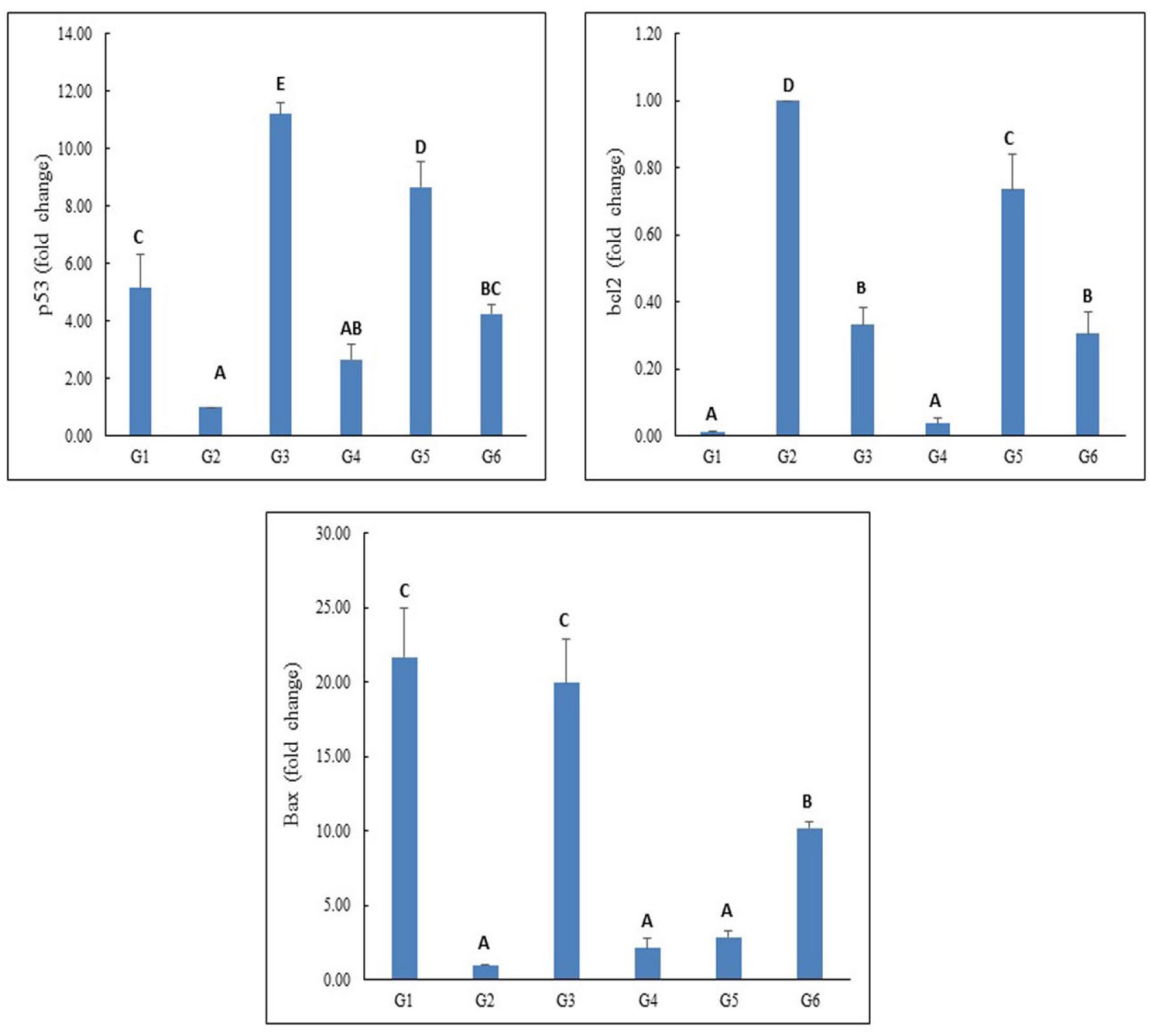
The expression levels of P53, Bcl2 and Bax genes in the tumor control group (G2), doxorubicin-treated group (G3) and groups treated with 100, 200 or 400 mg/kg of PSO nano-emulsion (G4, G5, G6) respectively. Data is displayed as mean ± standard error. Means marked with the same superscript small letters are insignificantly different (*p* > 0.05), whereas those marked with different ones are significantly different (*p* < 0.05).

**Figure 9:**
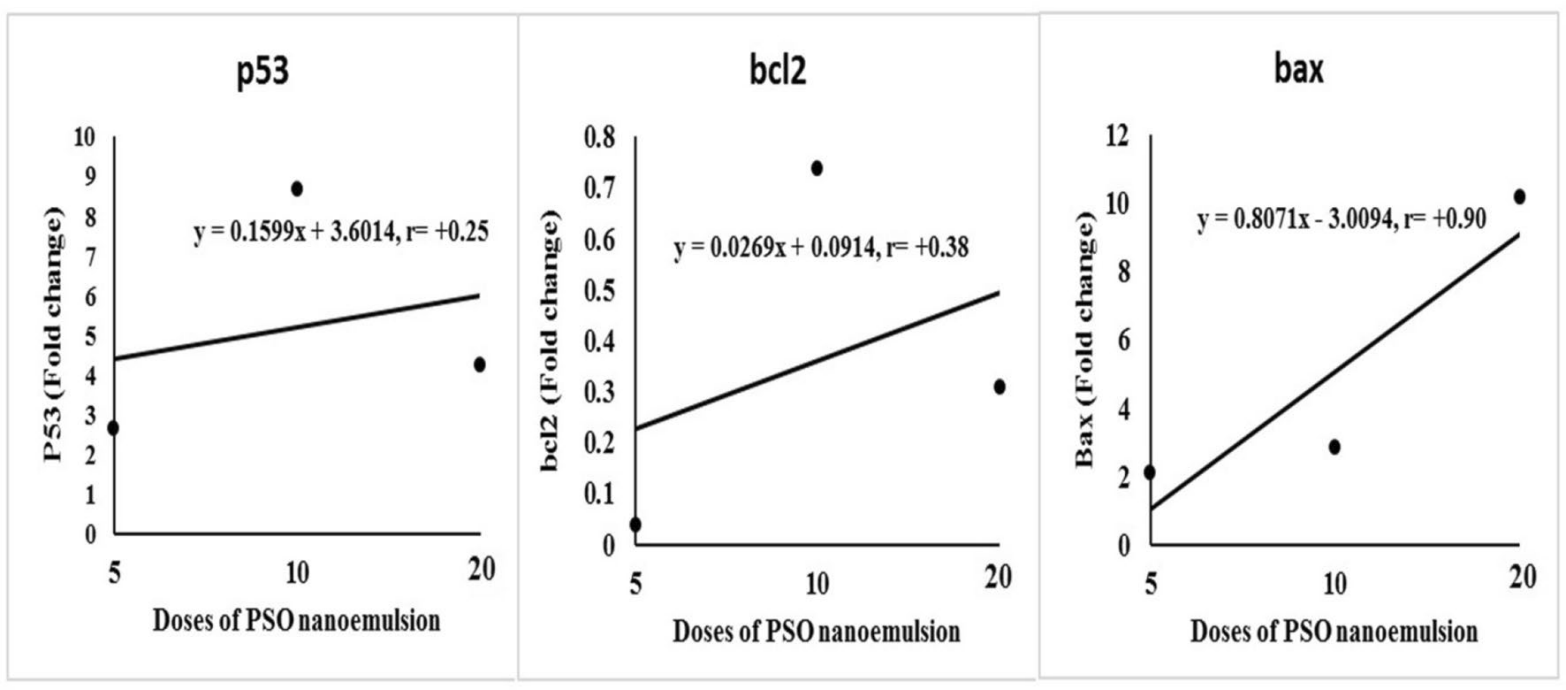
Regression analysis to illustrate the relationship between the different doses of PSO nano-emulsion and the expression levels of the studied p53, Bcl2 and Bax genes. r: Pearson’s correlation coefficient.

The expression levels of p53, Bcl2 and Bax genes are displayed in Figs. 8 and 9. Treatment with 100, 200, or 400 mg/kg body weight of PSO nano-emulsion (G4, G5, and G6 groups, respectively) resulted in a significant increase in the expression levels of p53 and Bax genes and a significant reduction in the expression level of Bcl2 gene compared to their expression levels in tumor tissues of tumor control (G2) group (Fig. 8). The expression levels of p53 and Bax genes were also remarkably increased in the tumor tissue of mice injected with doxorubicin (G3 group) compared to the expression levels of the tumor control G2 group. However, by giving the different doses of PSO nano-emulsion, the expression levels of p53 and Bax genes were substantially declined compared to doxorubicin group expression levels; whereas the expression level of Bcl2 gene revealed a significant decline in the G4 group treated with 100 mg/kg of PSO nano-emulsion, but marked elevation was observed in the tumor tissue of mice given 200 mg/kg of PSO nano-emulsion (G5 group) as compared to their expression levels in doxorubicin treated mice (Fig. 8).

Regression analysis and correlation coefficient evidenced a strong correlation between the expression levels of Bax gene and the tested doses of PSO nano-emulsion, whereas weak correlations were reported between the tested doses of PSO nano-emulsion and the expression levels of p53 and Bcl2 genes (Fig. 9).

### Histopathological examination

**Figure 10:**
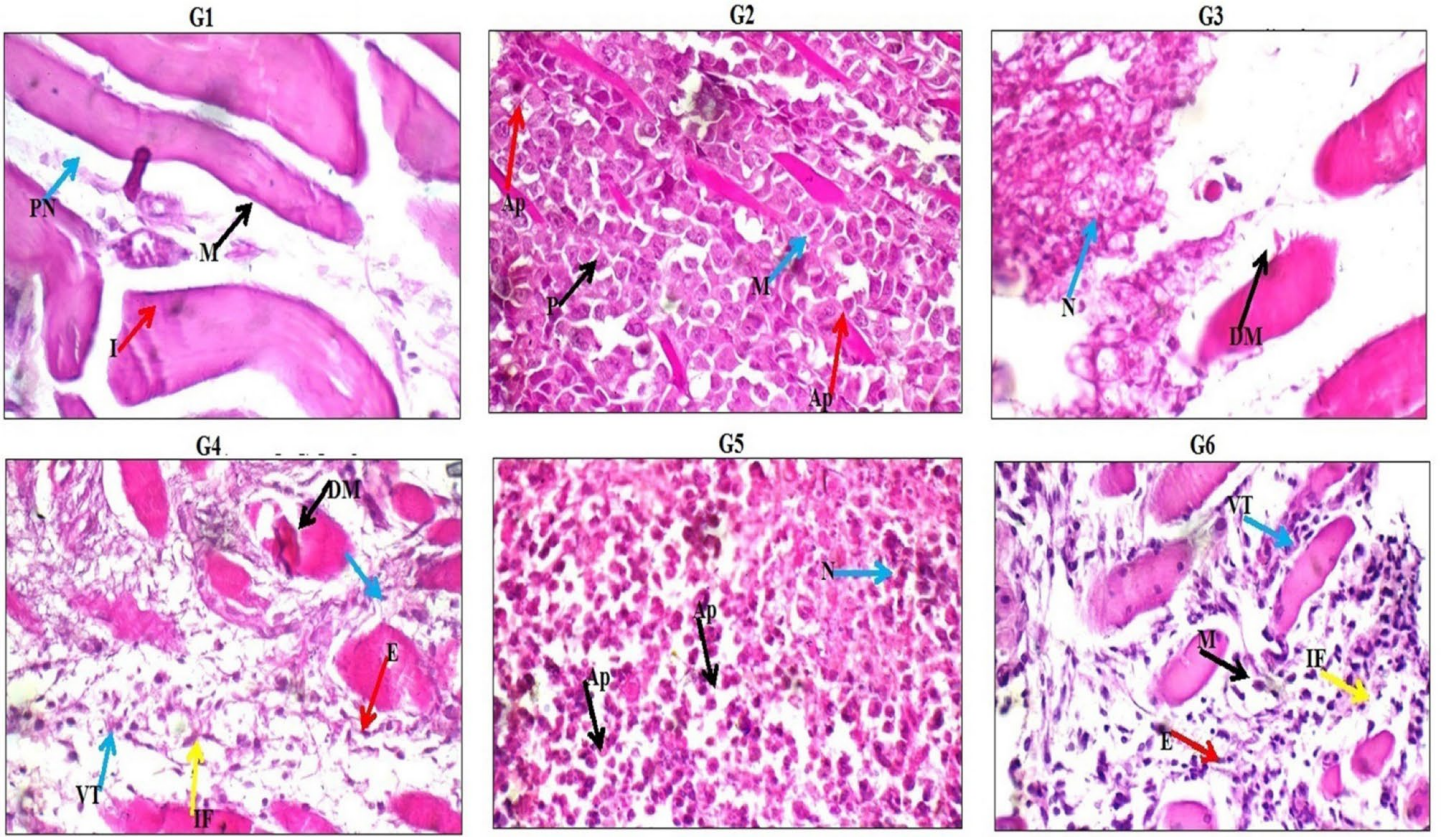
Histological examination of the tumor and muscle tissues of the negative control (G1), tumor control (G2), Doxorubicin treated (G3) and PSO treated (G4–G6) groups. DM: destructed muscle fibers; E: edema; VT: viable tumor cells; IF: inflammatory infiltrate; Ap: apoptotic cells; N; necrotic cells; M: skeletal muscle fibers; P: pleomorphic cells; PN: peripheral nuclei; I: interstitium.

Histological examination of the muscle and tumor tissues revealed normal muscle fibers with distinct borders and peripherally located nuclei in the skeletal muscles of healthy negative control group (G1 group). On the contrary, skeletal muscles of the tumor control group (G2 group) showed infiltrating tumor composed of sheets and nodules of markedly pleomorphic viable tumor cells with hyperchromatic nuclei infiltrating in between destructed muscle fibers with few scattered apoptotic cells, and small areas of necrosis (Fig. 10). Skeletal muscles of mice injected with Doxorubicin (G3 group) showed also markedly destructed and edematous muscle fibers and others with irregular indistinct cell borders and bright eosinophilic cytoplasm, and small area of markedly necrotic tumor tissue (Fig. 10). On the other hand, skeletal muscles of mice administered 100 mg/kg of PSO nano-emulsion (G4 group) showed mildly destructed muscle fibers with indistinct borders, mild interstitial edema with mild cellular infiltrate composed of viable tumor cells with scattered apoptosis and small areas of necrosis, and scattered inflammatory infiltrate (Fig. 10). Areas of edematous skeletal muscle fibers with marked interstitial edema and scattered inflammatory infiltrate, and infiltrating tumor composed of small nodules and sheets of viable tumor cells with marked apoptosis, and large areas of necrosis with karyorrhectic fragments were also seen in the mice given 200 mg/kg of PSO nano-emulsion (G5 group). In mice of G6 group that administered 400 mg/kg of PSO nano-emulsion, skeletal muscle fibers with marked interstitial edema and marked inflammatory infiltrate, mild peri-vascular edema, and small nodules and sheets of viable infiltrating tumor cells with scattered apoptosis, and large areas of necrosis with karyorrhectic fragments and small areas of hemorrhage were observed (Fig. 10).

### Immuno-histochemical localization

**Figure 11:**
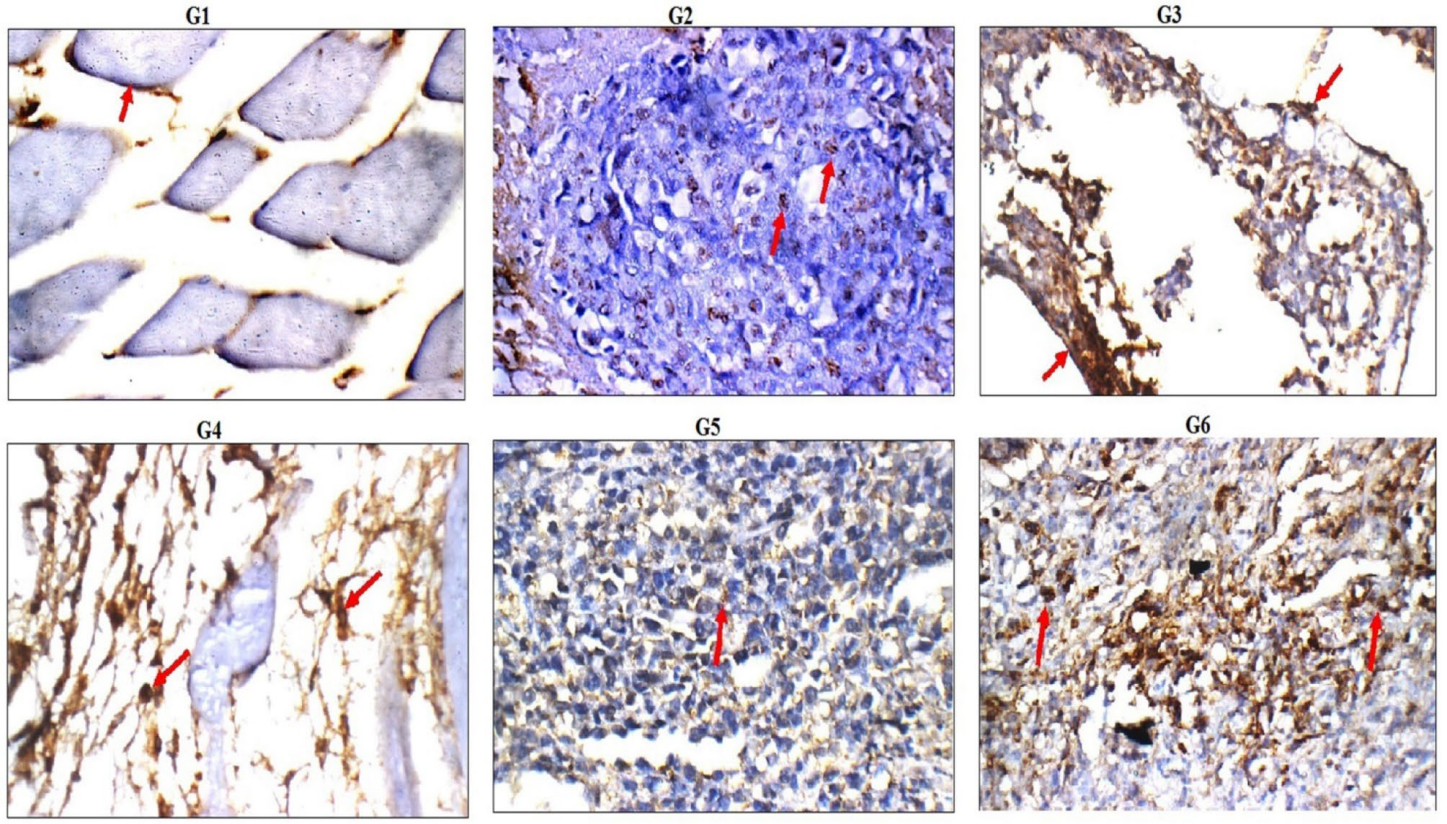
Immuno-histochemical localization of p53 protein (red arrow) in the tumor and muscle tissues of the negative control (G1), tumor control (G2), Doxorubicin treated (G3) and PSO treated (G4–G6) groups (p53 immunostain × 200).

**Figure 12:**
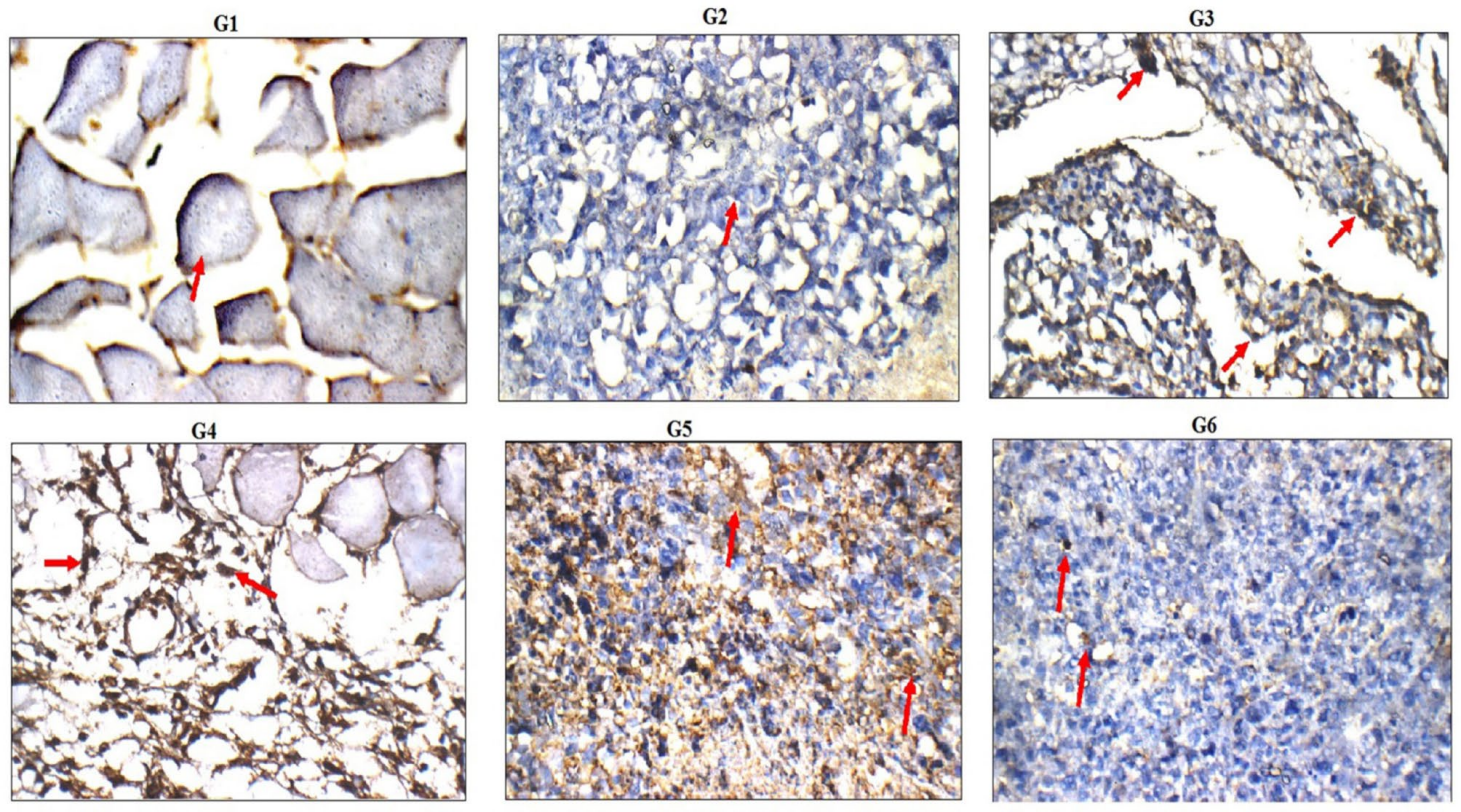
Immuno-histochemical localization of Caspase protein (red arrow) in the tumor and muscle tissues of the negative control (G1), tumor control (G2), Doxorubicin treated (G3) and PSO treated (G4–G6) groups (Caspase immunostain × 200).

Immuno-histochemical localization of p53 and Caspase proteins revealed negative reactivity of p53 and Caspase proteins in the muscles of healthy negative control group (G1 group), whereas, weak reactivity for p53 gene and negative reactivity for Caspase gene were observed in the tumor tissues of tumor control group (G2 group) as seen in Figs. 9 and 10. High accumulation of p53 and Caspase proteins were manifested by the seen high reactivity for p53 and Caspase proteins in the tumor tissues after Doxorubicin treatment (G3 group) and the tested doses of PSO nano-emulsion (G4, G5 and G6 groups) as displayed in Figs. 11 and 12.

### Oxidative stress markers

**Figure 13:**
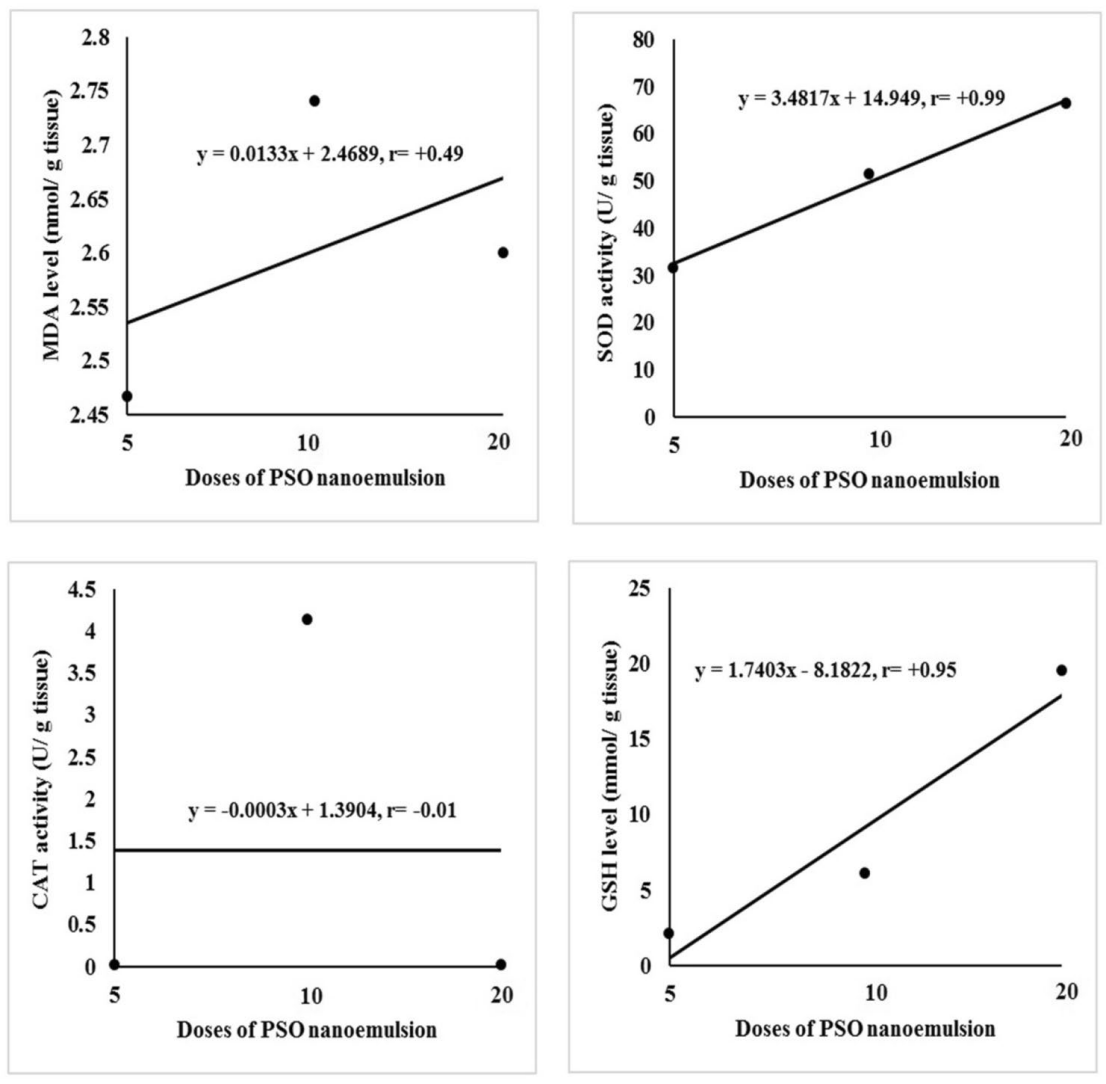
Regression analysis of the levels of MDA and GSH as well as the activities of SOD and CAT in the three groups administered different doses of PSO nano-emulsion (5, 10 and 20% of PSO LD50). r: Pearson’s correlation coefficient.

In Table 4 and Fig. 13, the levels of MDA and GSH and activities of SOD and CAT are recorded. The level of MDA level in the tumor tissues of mice injected with Doxorubicin (G3 group) or PSO nano-emulsion (G4, G5 and G6 groups) non-significantly changed compared to its level in the tumor control group (G2 group). Conversely, SOD activity was remarkably decreased in mice given 100 mg/kg of PSO nano-emulsion (G4 group) compared to the remaining four groups.

**Table 4:** The level of malondialehyde (MDA) and reduced glutathione (GSH) as well as the activities of superoxide dismutase (SOD) and catalase (CAT) in the normal negative control group (G1), tumor control group (G2), doxorubicin-treated group (G3) and groups treated with 100, 200 or 400 mg/kg of PSO nano-emulsion (G4, G5, G6) respectively.

Parameter	G1	G2	G3	G4	G5	G6
MDA(nmol/g)	1.65 ± 0.33^A^	2.53 ± 0.03^B^	2.77 ± 0.03^B^	2.53 ± 0.03^B^	2.74 ± 0.01^B^	2.53 ± 0.03^B^
SOD (U/g)	118.23 ± 11.82^C^	70.83 ± 10.99^B^	74.25 ± 7.55^B^	31.57 ± 7.70^A^	51.33 ± 8.23^AB^	66.39 ± 5.77^B^
GSH (mmol/g)	1.33 ± 0.18^A^	1.41 ± 0.40^A^	8.19 ± 1.56^B^	2.10 ± 0.76^A^	6.07 ± 0.44^B^	19.50 ± 1.80^C^
CAT (U/g)	9.37 ± 0.15^C^	0.04 ± 0.02^A^	0.21 ± 0.03^A^	0.02 ± 0.003^A^	4.13 ± 0.03^B^	0.01 ± 0.001^A^

Data is displayed as mean ± standard error.

Means marked with the same superscript letters are insignificantly (*p* > 0.05) different, whereas those marked with different ones are significantly (*p* < 0.05) different.

In the mice of G3 group, the GSH content was similar to that of the mice treated with 200 mg/kg of PSO nano-emulsion (G5 group) and was significantly higher than those in the G1, G2 and G4 groups, but markedly lower than the content of G6 group treated with 400 mg/kg of PSO nano-emulsion. The activity of CAT was substantially elevated only in mice given 200 mg/kg of PSO nano-emulsion (G5 group) and did not change significantly in G3, G4 and G6 groups compared to its activity in the tumor tissue of tumor control group (G2 group) as shown in Table 4. Strong relationships were observed between the tested doses of PSO nano-emulsion and the GSH content as well as SOD activity (Fig. 13).

## Discussion

The promising antioxidant and cytotoxic activities of PSO in different cancer cell lines along with the unique physicochemical properties and kinetic stability of nano-emulsion compared to its bulk materials have directed us to investigate the possibility of using PSO nano-emulsion as an alternative anticancer drug for the used traditional chemotherapeutic drugs used with low or no toxic side effects in the current study.

Monitoring of live ESC bearing mice demonstrated the potent effects of PSO nano-emulsion in tumor treatment and retardation as manifested by the high dose-dependent reductions in the tumor weight and volume observed after treatment with PSO nano-emulsions different doses. These results supported the previously demonstrated cytotoxic and anticancer effects of PSO in various experimental models^17–20^.

The demonstrated antitumor effects of PSO nano-emulsion in this study may be resulted from inhibition of tumor cells proliferation and induction of apoptotic death. Apoptosis, a programmed cell death, has distinctive morphological features and energy-dependent biochemical mechanisms. Apoptosis is a vital component of various processes including normal cell turnover, proper development and functioning of the immune system, hormone-dependent atrophy, embryonic development, and chemical-induced cell death. The therapeutic potential of many chemicals depends on its ability to modulate cell life or death^40^.

There are several extrinsic and intrinsic signals for apoptosis. For example DNA breaks act as a signal of apoptotic damage^41–44^. Our finding of significant elevations in the Comet parameters confirmed induction of DNA breaks by different PSO nano-emulsion doses in the tumor tissues and thus PSO nano-emulsion induced DNA breaks triggered apoptosis of tumor cells.

DNA breaks particularly double-stranded DNA breaks are one of the most lethal and dangerous types of DNA damage since one double-stranded DNA break is sufficient to destabilize the genetic integrity of the cell until it is killed^45–47^. Alkaline comet assay is very sensitive for detecting single and double DNA breaks^32^.

The induction of DNA breaks also stimulates apoptosis of tumor cells by inducing p53 gene activation^48^. The DNA damage response is triggered by the detection of DNA lesions. This response consists of an orderly sequence of signal transduction events that can induce the accumulation of p53, which plays a critical role in responding to various stresses that cause DNA damage, especially reactive oxygen species as differential phosphorylation modulate its stability as well as induction of its downstream gene products^49–51^.

Consequently, the antitumor effects of PSO nano-emulsion observed in this study can also be attributed to the simultaneous dose-dependent upregulation of the expression levels of p53 and Bax apoptotic genes and the reduction in the expression level of the anti-apoptotic Bcl2 gene noticed in tumor tissues of mice treated with PSO nano-emulsions because overexpression of the tumor suppressor P53 and Bax genes stimulates apoptosis^52^.

Apoptosis was further confirmed in this study by the high accumulation of the tumor suppressor p53 and Caspase proteins observed in the tumor tissues of mice given the tested doses of PSO nano-emulsions. Histological examination of the tumor tissues also manifested apoptosis and tumor regression after administration of PSO nano-emulsion through the appearance of apoptotic and necrotic cells and high infiltrations of tumor tissues with inflammatory cells. These results are consistent with the ability of normal-sized PSO to induce apoptosis in tumor cells demonstrated in previous studies^17–20^.

SOD and CAT enzymes are among the endogenous antioxidant enzymes that play a pivotal role in the elimination of superoxide radicals and hydrogen peroxide, thereby disrupting the proliferation of lipid peroxidation reactions and acting as protectors that protect against ROS-induced oxidative damage^53^. CAT is an oxidoreductase enzyme, which transforms H_2_O_2_ into H_2_O and O_2_ and protects cells from damage induced by ischemia reperfusion by scavenging ROS. SOD is antioxidant enzyme that protect the cells against reactive oxygen radicals mainly superoxide radicals by catalyzing the dismutation of two superoxide radicals to O_2_ and H_2_O_2_^54^.

Additionally, the functional groups of SOD (NH2 and -amino groups of lysine and SH groups of cysteine) are highly susceptible to oxidative damage. The conversion of SH groups to disulfides and other oxidative species (e.g. oxygen) is one of the early events observed during radical-mediated oxidation of proteins^55^.

Consequently, our finding of significant reductions in the activities of CAT and SOD enzymes observed in the tumor tissues of mice given PSO nano-emulsions manifested the disruption of the antioxidant defense system and damage of tumor tissues by PSO-nano-emulsion.

Regarding the safety of PSO nano-emulsion, slight variations in Comet parameters were observed in the liver and kidney tissues of mice given 100 or 200 mg/kg of PSO nano-emulsion until the %DNA in tail and tail moment became remarkably lower than that in the Doxorubicin injected mice. These non-genotoxic effects demonstrated in this study for low doses of PSO nano-emulsion are consistent with the study by Ferreira et al.^28^, that demonstrated the safety of PSO nano-emulsion for human blood cells.

However, treatment with the highest dose of PSO nano-emulsion (400 mg/kg) caused significant elevations in the DNA damage markers in the liver and kidney tissues, that makes the two low doses of PSO nano-emulsion (100 and 200 mg/kg) better for treating tumors than the highest dose. Meanwhile, histological examination demonstrated damage of liver and kidney architecture in mice give the three tested doses of PSO nano-emulsions as manifested by apoptotic cells, congested vein, dilated vein, inflammatory cells infiltration and loss of renal brush borders in liver and kidney tissues.

## Conclusion

Based on the data discussed above, treatment with tested doses of PSO nano-emulsions (100, 200 and 400 mg/kg) significantly reduced tumor volume and weight, in a dose dependent manner and induced DNA breaks that stimulates apoptosis of tumor cells by increasing the apoptotic p53, Bax and Caspase genes expression and decreasing the anti-apoptotic gene Bcl2 expressions. However, the highest dose of PSO nano-emulsion (400 mg/kg) caused DNA damage in liver and kidney tissues, thus, PSO nano-emulsion with a dose level of less than 400 mg/kg is recommended as an alternative antitumor drug for chemotherapy and more studies are required to shed more light on the mechanism of action of PSO nano-emulsion.

## Data Availability

The datasets used and/or analyzed during the current study are available from the corresponding author on reasonable request.
